# Formation of Nurses at Universidad de Antioquia: A Reality embodied in its Study Plans

**DOI:** 10.17533/udea.iee.v38n3e04

**Published:** 2020-11-11

**Authors:** Elvigia María Posada Vera

**Affiliations:** 1 Nurse, Ph.D. Professor, Facultad de Enfermería, Universidad de Antioquia, Medellín, Colombia. Email: emaria.posada@udea.edu.co Universidad de Antioquia Universidad de Antioquia Medellín Colombia emaria.posada@udea.edu.co

**Keywords:** education, nursing, curriculum, students, nursing, educación en enfermería, curriculum, estudiantes de enfermería, educação em enfermagem, currículo, estudantes de enfermagem

## Abstract

The article presents the trajectory of the study plans that have guided the formation of nursing professionals at Universidad de Antioquia in Medellín (Colombia). It presents the principal milestones or events that at a given moment determined significant ruptures in the formation processes and which show how this Faculty of Nursing, in its 70 years of existence, has always articulated the health needs of the context with the formulation of its study plans, with the sole purpose of forming critical and innovative nursing professionals capable of responding assertively to care needs, according with the principal challenges or trends that condition the health environment.

## Resumen

El artículo presenta la trayectoria de los planes de estudio que han direccionado la formación de los profesionales de enfermería en la Universidad de Antioquia de Medellín (Colombia). En él se presentan los principales hitos o acontecimientos que en un momento dado determinaron rupturas significativas en los procesos de formación, y que muestran como esta Facultad de Enfermería, en sus 70 años de existencia, siempre ha articulado las necesidades de salud del contexto con la formulación de sus planes de estudio, con el único propósito de formar profesionales de enfermería críticos e innovadores, capaces de responder asertivamente a las necesidades de cuidado, de acuerdo con los principales retos o tendencias que condicionan el entorno de la salud.

## Resumo

O artigo apresenta a trajetória dos planos de estudos que orientaram a formação dos profissionais de enfermagem da Universidade de Antioquia em Medellín (Colômbia), separados por marcos ou eventos que em determinado momento determinaram rompimentos significativos nos processos de formação, e que mostram como esta Faculdade de Enfermagem, nos seus 70 anos de existência, sempre articulou as necessidades de saúde do contexto com a formulação dos seus planos de estudos, com o único propósito de formar profissionais de enfermagem críticos e inovadores, capazes de responder assertivamente às necessidades de cuidado, considerando os principais desafios ou tendências que condicionam o ambiente de saúde.

## Introduction

Evidencing the trajectory of the study plans of the Faculty of Nursing at Universidad de Antioquia, Medellín (Colombia) is the common thread in this work, with the idea of facilitating the comprehension of the formation process experienced in its 70 years of existence and, hence, the development the profession has had in it. This process has been permeated by cultural, socioeconomic, political, and scientific-technical factors characteristic of each period, as well as by the characteristics of the profession’s internal development, which influenced, over time, the philosophies of the academic program and, consequently, the study plans, the pedagogical model and, in general, the curricular issues on which the formation of its students has been supported. In this sense, this article shows the principal milestones that have guided the study plans of this faculty and emphasizes the curricular transformations that derive from profound processes of reflection and permanent self-evaluation.

As pioneer in the formation of nursing professionals in the region, today - after 70 years - the Faculty continues with the commitment of preparing highly qualified professionals to satisfy the care needs of a society in permanent change. The trajectory described is not shown as a finished process; on the contrary, it leaves it open to permanent reflection to ensure the construction of pertinent and contextualized academic proposals that favor the positioning and visibility of nursing within the setting.

## Beginnings of the formation 

The need to train nurses in Medellín became evident since early 20th century, however, only until 1950 was the first school of nursing created, under the direction of the religious community of the Sisters of the Presentation, whose scientific knowledge and technical capacity were backed by Universidad de Antioquia.

As with other institutions preparing nurses at the time in the country, the study plans had a three-year duration and granted the general nursing degree, which would today correspond to a technological formation. The first study plan was constituted by an offer of basic assignments, with eminently biological foundation, whose contents were determined by the professors of the time, who were mostly physicians, leaving them to decide the knowledge the nurses had to learn. In turn, the religious instructors had to strengthen in the students the skills and procedural abilities to care for patients, under a technical and instrumental logic. Memorization was the fundamental learning method that demanded acritical, silent, and obedient acceptance, which was assimilated through repetition, without including any reflective or analytical element and - much less - of discernment and critique upon such.(1)

Skills, procedural ability, and attitudinal and affective components were the most relevant aspects of the formation process during this time. For the religious, emphasis was placed on training the personnel in passive compliance of the norm, order, discipline, and absolute obedience of the entities considered of superior order, like physicians and priests, values in keeping with their religious principles and the invisible and subordinate role assigned to women. This particular way of thinking, reasoning, and acting was gradually implemented in the formation of the students, amid learning lacking disciplinary and philosophical conceptualizations and by means of thoughtless repetition of theory and practice, within the disciplinary framework of pastoral power(2) that shaped their subjectivity, where everything was assimilated as something natural and non-problematic.

Parallel to this formation experience, the offer of health services in the country was influenced by the Flexner model, which grouped patients according to type of diagnoses, classifying them by specializations, under the premise of considering the human body as a set of systems disconnected from each other, isolated from its social environment, which is why disease was seen as an aggregate of signs and symptoms, whose treatment was similar independent of the person. This model was implemented in Colombia, following recommendations by the North American medical missions that visited the country in the early 1950s and was determinant for the adoption of an education model in medical schools throughout Latin America and, through these, in the schools of nursing.(3) This was how the formation of the health staff, and in this case nurses, placed disease at the core, with a predominantly curative practice that considered the hospital as its principal scenario.

The strength of the Flexner proposal, added to poor conceptual development nursing had in Colombia, strengthened the positivist paradigm in the theoretical and practical formation imparted in the school during the early 1950s, with technical-scientific foundation exclusively biological and organicist and an ordering of the study plan supported on the scheme of medical specializations, with which the distorted imaginary of the nurse was strengthened, as an aide to the physician.(4) Although the study plan of the School of Nursing at la Universidad de Antioquia had its first modification by late 1950s, this obeyed more the need manifested by the students of diminishing their academic load, than the scientific, curricular, or pedagogical developments that led to a change in its orientation.(5)

## Approach to public health as a new reality

During early 1960s, the country posed the political need to plan health and education in centralized manner, articulating them the planning of socioeconomic development. This was a response to the orientations by the Inter-American Economic and Social Council and by the Organization of American States, which in the country took shape in the so-called Alliance for Progress, seeking to respond to the political, economic, and social problems in Latin America, given the region’s social inequality and the mandates characteristic of the cold war. For the country, this implied stopping the potential influence of the socialist movement facilitated by the success of the Cuban revolution in 1959.(6)

The formation of the health staff was one of the most relevant issues in Colombia's planning intention, in relation with the assignment and use of resources. Specifically for nursing, characterization studies were begun, which evidenced that the highest percentage of graduate nurses was concentrated in capital cities, the majority in the hospital area, and very low percentage in outpatient services.(7) These results showed the importance of redirecting nurses toward the rural area, which led to the idea of implementing the compulsory social year immediately after the students finished their studies, which, additionally, would permit broadening the health care coverage. Moreover, as part of the planning policy promoted by the United States, the Inter-American Cooperative Public Health Service was created, aimed at technical and financial support to train technicians in public health. With these means, the technical guidelines were oriented and defined to face the main problems in this area, from a hygienist conception,(6) which considers disease a consequence of exposure to inadequate environmental conditions that can be corrected hygienic means.

This policy influenced decisively on the orientation of the formation and exercise of the nurses, during a period in which an important number of international advisory consultants, especially North American, visited institutions forming nurses in the country, including the School of Nursing at Universidad de Antioquia. Simultaneously, training of nurses in the United States was favored, through scholarships granted by international health organizations, like the Pan-American Health Organization and foundations, like the Rockefeller and Kellogg foundations.(6)

By this time, the revision of the study plans from the schools of nursing in Latin America, conducted by a group of nursing professionals supported by the World Health Organization, evidenced the lack and/or marginality of contents related with public health, transmissible diseases and psychiatry and, in turn, highlighted as the principal flaw the inadequate formation of nurses for their performance in professor and administrative functions.(5) This was reaffirmed in the 1961 Seminar on Nursing Education held in Paracas, Peru; as of that moment, the professor role was included as part of their functions.(7) For the School of Nursing at Universidad de Antioquia, this implied a rupture in the orientation toward training in technical procedures to meet the demands of the medium, which the graduates already manifested openly.

Thus, in 1963, the study plan was revised and a new proposal was aired, which incorporated and strengthened public health and mental health, adjusting the contents to the hygienist trends of health care. Public health acquired a leading role, formation was aimed at strengthening skills in actions of health prevention and recovery, supported on epidemiology and biostatistics, which later was reflected on the institution’s academic and research production. Mental health began to glimpse a turn toward the social and community.(8)

By 1965, following the orientations of the Colombian Association of Faculties of Nursing (ACOFAEN, for the term in Spanish) the complementary program of the Nursing degree was created. This implied offering an additional proposal to the three-year technological formation, which strengthened areas of administration, teaching, with research elements, including new orientations of caring for healthy individuals, with a preventive look where disease continued being the center. This new orientation toward caring for the healthy individual, necessarily led to adopting new pedagogical strategies for the teaching-learning process, propitiating the development of extramural teaching models. The degree in Nursing accredited professionals to manage nursing departments and work with health and education programs in the area, which also gave free access to graduate studies, like specializations and master’s.(5)

## Enhancement of the community practice

During the 1970s, the economic crisis in Latin America obligated countries to reduce drastically public expense, with disastrous consequences for social services and among them health services, which is why it was necessary to adopt health systems that, in spite of being more rational, permitted achieving universal coverage. With this intention, the World Health Organization and the World Bank prioritized programs in Primary Care or Basic Health Services that used simple and affordable procedures that enabled the maximum use of scarce resources available.(6) 

In coherence with this orientation, Colombia broadened health coverage to the least protected areas and the compulsory social service was institutionalized. In 1977, it was legalized as a requirement, which justified in the School of Nursing implementation of the rural practice of students in senior year.(5) Contact with the social reality of the communities was a fact that transformed the vision on the origin and care of health and disease, upon understanding that these phenomena were closely related with the sociocultural, political, and economic contexts of the people, which explained their complex and multidimensional nature, not reducible to exclusively biological or physiological explanations, or to the exploration of isolated and circumstantial phenomena.(4)

This comprehension stirred great interest in the study of the existing relation between disease and society, a stance assumed by the interdisciplinary movement of social medicine in Latin America.(4) Several professors from the School of Nursing had a leading role in the gestation and development of this movement in Colombia and specifically in Medellín through a group led from the National Faculty of Public Health at Universidad de Antioquia, which managed to energize academically the school with profound reflections that showed the need to transcend from a merely clinical, individual, biological, and positivist vision to nursing practice with greater critical conscience and an increasingly broader and humanistic vision of health and of the social context.

This panorama revealed the urgency for a curricular reform in the general nursing program and in the degree program. On one side, recognition and increasing acceptance of the social current in the health area and its questions of the conceptual and methodological bases that guided the offer of services, favored the entry of new more comprehensive and encompassing concepts; and on another, the implementation of new policies in the health and education areas, related with broadening the coverage, strategies of primary care, and community participation. Thus, these favored areas, like public health, mental health, social medicine, environmental sanitation and health administration. As response to this need, after multiple debates, the proposal was consolidated of a new study plan that sought to transcend the clinical, individual, biological, and positivist view toward a more social conception of the nursing practice. A new conceptual framework guided the formation in the school, giving way in care to clinical care, as well as to the social and the collective.

Based on the natural and social history of disease, this new study plan was oriented by levels of growing complexity, considering the healthy or sick individual in his social and community life, under a concept of disease prevention, treatment, recovery, and rehabilitation.(5) Explicitly, recognition is granted to the four performance areas nursing professionals have: care, teaching, administrative, and research. In this sense, students begin to recognize themselves as active and purposeful subjects; and professors as counselors and guide. Thus, entered the concept of co-responsibility with great relevance, which modified the teaching-learning strategies. Responding to an integrating conception, the change of the study plan in 1978 sought greater articulation through the establishment of courses from basic sciences, epidemiology, nutrition, administration, and mental health, as transversal to the academic program.(5) The new study plan, approved amid resistance and fear of abandoning the traditional schemes and opening the mind to new concepts and methods favored the creation of a unique four-year program leading to the Nursing degree, approved in 1980 by the Colombian Institute for the Promotion of Higher Education (ICFES, for the term in Spanish). Consequently, in May 1981, the Superior Council at Universidad de Antioquia approved the transformation of the School into Faculty. (5)

## Enhancement of knowledge

At the start of the 1980s, the Faculty of Nursing establishes for the first time a professional profile that guided the formation of its students, in compliance with the exigency by the ICFES as condition to certify the program. Reflecting on this profile permitted identifying new areas in which the nursing profession could start to dabble, bearing in mind the possibilities and care needs in the environment, emphasizing on primary health care and in areas, like sports medicine, occupational health and in pre-school, scholar, and adolescent care programs, and care for the elderly. Furthermore, complementing the care, administrative, teaching, and research functions it could perform in the clinical and outpatient areas. This generated a new curricular proposal, approved by ICFES in 1984, with a philosophy and objectives not very different from the program approved in 1980.(5)

Training and qualification opportunities, on the rise for professors from the Faculty of Nursing during the 1980s, were key to strengthen research and the approach to the development of the disciplinary discourse, which had been advancing in other countries around the world, where there was already talk of the importance of the conformation and appropriation of a body of knowledge characteristic of nursing, of a model of conceptual orientation and of innovation and expansion of a new professional role. This secured the idea of implementing and assuming new concepts, besides theories and models in nursing, which impacted the undergraduate study plan and later pushed the creation of graduate programs. A fundamental change was the organization of the study plan outside the medical specializations that till then guided its nominations, to assume the vital cycle as the tracer axis, for example, from obstetrics Gynecology, it went to caring for the mother and child, from medical-surgical to caring for the adult and so on. Besides breaking with the nominations derived from the medical model, the aim was to make explicit the social responses nursing had to provide regarding the health-disease process.(5) By the end of the 1980s, teaching of nursing demanded greater social and political awareness that placed the profession in more autonomous, purposeful, and reflective planes against the social, institutional, and disciplinary needs, contemplating central elements in the formation, like comprehension and strengthening of the disciplinary discourse, continuous formation, professional autonomy, interdisciplinary work, and research as essential source of knowledge.

## Complexity of a normative context that redirects the practical exercise of nursing

In 1991, a new Political Constitution was agreed upon for the country, with a perspective of democratization of the State and society, with political, ideological, regional, religious, ethnic, and cultural plurality. Legislation 30 of 1992 established a new philosophy of education, promoting human development through formation processes that stimulate high participation, responsibility, autonomy and self-criticism by the students. All these guided institutions toward a more integral formation, considering the person in its totality, not only circumscribed to the professional development. This new vision required more participative pedagogical models that recognized the capacity of the subjects from their overcoming the limits of automatic repetition of knowledge and lack of professional judgment.

At the start of this decade, the country’s health sector underwent an important reorganization in its normativity, with effects on nursing formation and its practice. The proposal founded the social development in the market, without losing sight of the increase in health level with strategies that permit enhancing a health culture, based on promotion and prevention to, thus, improve the efficacy of the sector. The reorganization of the health system was based on the decentralization of health services, assigning responsibility to each municipality and, more specifically, to its maximum health authority from its own territory, which is why effective participation by the community was essential. This proposal increased requirements for human resources in health in the first levels of care, with notable strengths in management, social participation, health promotion, and disease prevention.(7)

Paradoxically, Legislation 100 of 1993 was characterized by a marked trend toward the privatization of health, under the logic of free enterprise, within the neoliberal reference framework and reduction of the economic intervention by the State. The possibilities that the new constitution granted to the private sector to open their health programs placed free competition on the scene and, from there, the dilemma of competing with quality in providing the service and in programs of human resource formation. Within this scenario, during the early 1990s, the Faculty of Nursing saw a new process of analysis and self-evaluation of the study plan, having as inputs the regulatory scenario, the demands of the University to maintain updated study programs, progress in the scientific foundation of the nursing discipline, and the trends that showed nursing in an important moment of disciplinary and professional transition. According to Velandia,(7) these *trends comprise changes that range from the biological to the bio-psychosocial, from the individual to the group, from the curative to prevention, from the individual to community, from the hospital to ambulatory, from hierarchical to participatory, from the centralized to decentralized, from the quantitative to qualitative, from results to processes, from the regional to interregional, from single disciplinary to interdisciplinary, from sectorial to inter-sectorial, from manual management of data to computation, from closed information to broad and open communication. (p. 168)*

After many debates, in the Faculty of Nursing it was agreed to go from the health-disease concept to that of care, as tracer in the vital cycle, upon recognizing care as the object of study by nursing and upon adopting the Nursing Care Process as the methodology *par excellence* and the models and theories of nursing as sources of guidance. Similarly, it was considered that the history of nursing had to be part of the study plan to contextualize care within the development of the profession.(5)

The formation of the subject was assumed as a relevant issue, recognizing the importance of training in values, like respect, tolerance, participation, democracy, freedom, autonomy, development of self-esteem, and criticality among others, which had to be developed and strengthened in par with formation in *doing and knowing.* From the pedagogical, the need was recognized to adopt a more dynamic, flexible, and inclusive model, where students are leading players in their formation process and professors counselors who encourage participation and autonomy. The thematic contents of the courses were selected according to the profile of the alumni and the theoretical-practical integration was promoted seeking to favor learning and relevance was granted to the cognitive and affective goals throughout the formation process, which is why reproduction and repetition of information was left behind as a way of approaching knowledge.(5) Emphasis was placed on integral human development by using participation strategies that would favor the concurrence of all knowledge.(9)

This curricular proposal, made official in 1998, established in its conceptual framework various central categories, like the human being, nursing, nursing care, environment, the human vital process, and learning environments in which nursing care became the central axis around which revolves the formation process, supported on the concepts and theories of nursing, specified in the development of practice in the clinical and community setting.(9)

These elements support the curricular structure from the basic foundation line, constituted by knowledge from different sciences or disciplines that provide to students a cognitive support to access and comprehend more complex processes; likewise, from the professional-disciplinary foundation line, where knowledge is acquired characteristic of nursing as discipline and as profession (theories, concepts, methodologies, among others), considering the individual clinical and social community dimensions in the professional exercise; and, finally, the deepening line in which management is delved into as a specific area of this profession. The curricular reform of 1998 maintained the four-year formation, which sought to develop in the students, academic and professional skills within the roles of caregiver, researcher, manager and educator,(9) which led in 1999 to obtaining for the first time the Accreditation of Quality from the National Ministry of Education for five years, a process repeated later in 2006 for seven years and in 2013 for eight years.

## The first two decades of the 21^st^ century: care needs in a globalized world

Since the first institutional accreditation, the Faculty of Nursing entered a permanent process of self-evaluation of the framework of norms that regulate health and education, but also of the changes and exigencies of an increasingly complex and dynamic context. These evaluations have led to adjustments and modifications in the curricular structure, without undertaking a radical reform. Since 2008, adjustments have been made on the flexibility and social pertinence of the study plan, and in this measure, it has been updated according to specific needs. Currently, work in underway on the fifth curricular plan version, approved since 2017 by the National Ministry of Education. Adjustments during these two decades of the nascent century account for the necessary reflection regarding the pertinence of its study plans to prepare in a globalized world nursing professionals that can respond to an epidemiological profile in transition, to demographic transformations, and to the big social problems that advocate for intersectoral and interdisciplinary work. Inter- and trans-cultural care and social conscience and sensitivity are today key issues in the formation.

Since 2010, the competencies approach was adopted in the formulation of courses from the orientations made by the University, which led to the question of the relevance, articulation, and integration of content and of the methodology employed in the teaching-learning process. Added to this, the results of the program’s self-evaluation, conducted in 2012, were key in raising awareness of the need for a more radical change in the faculty’s curricular structure. They highlight the lack of indispensable knowledge in the profile of the current nursing professional, like health and mental ailments, care related with old age and aging, strengthening in knowledge and skills in basic nursing procedures, education for health, and political formation, without discarding the need to delve into the family and community health area and continue strengthening management and research. These needs were established in the diagnosis of current care needs performed by the academic community in 2016, when this new reform was decisively promoted.

This proposal of change considered as important element to review the profile of the alumni from the Faculty of Nursing at Universidad de Antioquia, in coherence with the principal challenges or trends proposed for the profession and which must be resolved with formation in the program, without leaving aside the University’s commitment to an integral formation, within a process of co-responsibility among the university, family, society, and the subject, which must involve in the formation, besides technical-professional issues, others of socio-humanist order.

The curricular structure proposed, which seeks to stimulate the integration, innovation, interdisciplinarity and interculturality, and flexibility as fundamental principles is grounded on an integrated pedagogical model, which according to Basil Berstein and Mario Díaz,(10) seeks for the diverse contents or knowledge to not go on different paths, but rather maintain an open relationship with each other. Its purposes include achieving the systemic articulation of knowledge, student-centered education, and significant learning, with emphasis on formative research.

The proposal is structured from three components: basic, disciplinary, and professional and linked to them, the socio-humanistic, investigative, and innovative and caring integrating nuclei and the academic nuclei in which knowledge is grouped into areas of knowledge or problems, like social, human, and care sciences, and formative research among others, and from here the thematic nuclei that make up the courses. Both the components and the nuclei are articulated together in the design and implementation of the courses.(11) From this route, the program’s formation purposes are deployed, in a relation of interdependence and not of hierarchy.

The new curricular structure is organized in a study plan that goes from 8 to 10 academic semesters. In it, research and innovation, health and mental ailments, and family and community health are configured as important nuclei, which were not visible in the prior study plan. Likewise, courses of basic and advanced nursing processes and procedures are integrated, as well as the course on aging and old age. From the pedagogical point of view, a model is proposed that highlights the participation of students in their learning process, which conceives the professor as a guide or advisor. Moreover, other teaching methods are strengthened, using the new communication and information technologies (CIT).

From the point of view of flexibility, a bank of elective courses is proposed that allows strengthening or enhancing the knowledge acquired in the compulsory courses, according with the specific interests of the students, which is also made visible through lifting the requirements and pre-requisites, free election of the emphasis in an area for the clinical professional practice and flexible research.

## Challenges

The new study plan seeks to go from a pyramidal approach where the areas of knowledge were divided hierarchically, (basic foundation, disciplinary foundation, and professional foundation), to an articulating structure that does not see knowledge in stages but complementary and in network, which - thus - permits make visible the need to establish methodologies that permit integrating and strengthening knowledge, from *being*, *knowing* and *knowing-how*, which gradually are acquired during the formation process, not as finished processes, but, on the contrary, open to change and to the transformations that the world imprints in their professional work.

Today, the characteristics of the context show us the need for a globalized study plan where interculturality is protagonist. In this sense, multilingualism is essential, as well as the increase of ICT as a pedagogical tool. All this favors the homologation of professional degrees; nevertheless, demanding - in turn - a large component of flexibility that considers a student that, besides being a worker, is a citizen of the world.

In addition, other academic processes are strengthened related with internationalization, cooperation, taking advantage of the existence of agreements with universities from other countries and regions, which permit two-way exchange of professors and students, membership to international networks of nursing and health and, from this logic, carry out academic events, multi-center research in addition to consulting and advising.

## Conclusions

As described at the beginning, this text has shown how in each period the characteristics and exigencies of the context, which with respect to the formation of the nursing professionals, is an aspect that has permeated their formation, which has been subject to political, cultural, social, academic, and economic inputs that have guided the objectives and philosophy of the program over time, its study plans and pedagogical models and, in general, the internal formation dynamic.

In that sense, the Faculty of Nursing shows that its development has been parallel with theoretical and epistemological discussions on health and disease, existing norms and all its endogenous development. Development of the faculty during these 70 years has taken place within a dynamic of changes and transformations of the society, with a permanent commitment to consolidate highly qualified training programs of nursing professionals, with capacity for constant transformation to respond critically and innovatively to the care needs of society during each period.
